# Validation of the International Medullary Thyroid Carcinoma Grading System in a Multicenter Cohort: Digital Image Analysis-Based Evaluation of Ki-67 Cutoffs and Assessment Methods

**DOI:** 10.1007/s12022-026-09940-0

**Published:** 2026-11-11

**Authors:** Milim Kim, Su-Jin Shin, Ji-Young Park, Chan Kwon Jung, Jang-Hee Kim, Soo Jin Jung, Jae Kyung Won, Hyeongchan Shin, Jin-Man Kim, Hee Jung Kwon, Jee Yeon Kim, Yeseul Kim, Ju Yeon Pyo, In Ho Choi, Lucia Kim, Hee Young Na, So Yeon Park

**Affiliations:** 1 Department of Pathology, Severance Hospital, Yonsei University College of Medicine, Seoul, Republic of Korea; 2 Department of Pathology, Gangnam Severance Hospital, Yonsei University College of Medicine, Seoul, Republic of Korea; 3 Department of Pathology, Kyungpook National University Medical Center, Daegu, Republic of Korea; 4 Department of Hospital Pathology, Seoul St. Mary’s Hospital, Seoul, Republic of Korea; 5 Department of Pathology, Ajou University School of Medicine, Suwon, Republic of Korea; 6 Department of Pathology, Inje University Busan Paik Hospital, Busan, Republic of Korea; 7 Department of Pathology, Seoul National University Hospital, Seoul, Republic of Korea; 8 Department of Pathology, Keimyung University Dongsan Hospital, Daegu, Republic of Korea; 9 Department of Pathology, Chungnam National University Hospital, Daejeon, Republic of Korea; 10 Department of Pathology, Gonyang University Medical Center, Daejeon, Republic of Korea; 11 Department of Pathology, Yeungnam University College of Medicine, Daegu, Republic of Korea; 12 Department of Pathology, Pusan National University Yangsan Hospital, Pusan National University School of Medicine, Yangsan, Republic of Korea; 13 Department of Pathology, Korea University Anam Hospital, Seoul, Republic of Korea; 14 Department of Pathology, Catholic Kwandong University International St. Mary’s Hospital, Incheon, Republic of Korea; 15 Department of Pathology, Soonchunhyang University Seoul Hospital, Seoul, Republic of Korea; 16 Department of Pathology, Inha University Hospital, Incheon, Republic of Korea; 17 Department of Pathology, Seoul National University Bundang Hospital, Seoul National University College of Medicine, 82, Gumi-ro 173 Beon-gil, Bundang-gu, Seongnam, 13620 Gyeonggi Republic of Korea

**Keywords:** Medullary thyroid carcinoma, IMTCGS, Ki-67 index, Digital image analysis, Hotspot, Prognosis

## Abstract

Although the International Medullary Thyroid Carcinoma Grading System (IMTCGS) was proposed to standardize the histologic grading of medullary thyroid carcinoma (MTC), the validity of its 5% Ki-67 index cutoff and the optimal assessment method remain to be fully established. This multicenter study aimed to validate the Ki-67 index component of the IMTCGS in a Korean cohort using digital image analysis (DIA) and to determine the most clinically relevant evaluation method. We retrospectively analyzed 187 surgically resected MTC cases across 16 institutions. Ki-67 indices were evaluated via DIA using two approaches: the hotspot method (highest proliferative area) and the average method (across three representative regions). Prognostic values of various Ki-67 cutoffs (5%, 3%, and ROC curve-derived thresholds) were evaluated independently and within the IMTCGS framework. IMTCGS high-grade status incorporating the 5% hotspot Ki-67 index emerged as an independent prognostic factor for recurrence-free survival (RFS), along with male sex, elevated postoperative serum calcitonin levels, and pN1b status. IMTCGS grading using the 5% average Ki-67 index failed to retain independent significance. While the composite high-grade IMTCGS classification remained an independent predictor of RFS in multivariate analysis, none of its constituent components (tumor necrosis, mitotic count, or Ki-67 index) retained independent prognostic significance when evaluated separately. This multicenter study validates the prognostic utility of the IMTCGS in Korean MTC patients. DIA-based hotspot Ki-67 assessment provides superior prognostic stratification compared to the average method, supporting its preference for routine clinical IMTCGS implementation.

**Supplementary Information:**

The online version contains supplementary material available at https://doi.org/10.1007/s12022-026-09940-0.

## Introduction

Medullary thyroid carcinoma (MTC) is a rare malignancy of parafollicular C cells that accounts for approximately 2–3% of all thyroid cancers and exhibits a highly variable clinical course [1, 2]. Although surgery remains the primary treatment modality, approximately 75% of patients with a palpable mass present with lymph node metastasis at diagnosis, and distant metastasis occurs in up to 10% of cases [3, 4]. Five-year and 10-year survival rates range from 75–96% and 64–91%, respectively, depending on disease stage [5–7].

Several clinicopathologic factors have been shown to be associated with patient outcomes in MTC, including tumor size, extrathyroidal extension, lymph node metastasis, distant metastasis, serum calcitonin and carcinoembryonic antigen (CEA) levels, and *RET* mutation status [6, 8–12]. However, despite its classification as a neuroendocrine neoplasm, MTC historically lacked a standardized histologic grading system—unlike pulmonary and gastroenteropancreatic neuroendocrine neoplasms (NENs), in which grading based on mitotic activity, tumor necrosis, and Ki-67 proliferative index is well established [13, 14]. More recently, however, grading based on these parameters has been shown to correlate with prognosis in MTC as well. Alzumaili et al. proposed a two-tier grading system based on mitotic count and necrosis [15], while Fuchs et al. developed a three-tier system incorporating mitotic count, necrosis, and Ki-67 index [16]. Building on these frameworks, the International MTC Working Group proposed the International Medullary Thyroid Carcinoma Grading System (IMTCGS) in 2022, which classifies MTC as high-grade if any of the following criteria is met: mitotic count ≥ 5/2 mm^2^, Ki-67 index ≥ 5%, or the presence of tumor necrosis [17]. Previous studies have validated the efficacy of the IMTCGS predominantly in Western populations [12, 18–22], whereas evidence from Asian populations is limited [23–25].

A critical issue within the IMTCGS framework is the assessment of the Ki-67 proliferative index. Although multiple studies have validated the prognostic utility of the IMTCGS, some variability exists in the methods used to assess the Ki-67 index across studies. Most studies adopted the protocol used for gastrointestinal tumors [13], counting 500 to 2000 tumor cells in the most proliferative area [17, 18, 20, 21], while Fuchs et al. primarily used tissue microarray (TMA) cores and counted a minimum of 100 tumor cells [16].

Despite the central role of the Ki-67 index in the IMTCGS, the recommendation for hotspot-based assessment rests primarily on expert consensus and extrapolation from other NENs rather than on direct evidence in MTC. Data directly comparing hotspot- and average-based Ki-67 assessment within the IMTCGS framework are largely lacking, although Fuchs et al. [16] reported that distinct hotspots were not observed in whole-slide sections and that Ki-67 indices assessed on TMAs correlated well with those in whole-slide sections. Therefore, it remains uncertain whether hotspot assessment offers greater clinical relevance than averaging across representative tumor regions. Furthermore, the validity of the currently recommended 5% cutoff has not been fully established in Asian cohorts. Digital image analysis (DIA) offers a standardized and reproducible approach for Ki-67 quantification and provides an opportunity to address these unresolved questions.

In this multicenter study involving 16 Korean institutions, we sought to validate the prognostic utility of the IMTCGS in a large Korean cohort of MTC patients. More importantly, we aimed to systematically compare hotspot- and average-based Ki-67 assessment methods using DIA to determine which approach provides superior prognostic stratification. Finally, we investigated the prognostic significance of alternative Ki-67 cutoffs to explore the optimal threshold for routine clinical application.

## Materials and Methods

### Study Population

This retrospective multicenter study included 187 patients with MTC who underwent surgical resection between January 2014 and December 2018 across 16 Korean tertiary institutions. MTC cases were initially identified through institutional pathology databases, and those with insufficient tumor tissue for Ki-67 immunohistochemical analysis were excluded. Clinical information—including patient age, sex, *RET* germline mutation status, preoperative and postoperative serum calcitonin and carcinoembryonic antigen (CEA) levels, surgical procedures, TNM categories, overall stage based on the 8th edition of the American Joint Committee on Cancer (AJCC) Staging Manual [26], adjuvant therapy, and follow-up data—was retrospectively collected from electronic medical records. The study was approved by the Institutional Review Board (IRB) of each participating institution, with a waiver of informed consent granted owing to the retrospective study design.

### Histopathological Assessment

Hematoxylin and eosin (H&E)-stained slides of all cases were reviewed by a designated endocrine pathologist at each participating institution. Evaluated pathological variables included tumor size, multiplicity, mitotic count per 2 mm^2^, tumor necrosis, nuclear pleomorphism, prominent nucleoli, spindle cell component, sheet-like growth pattern, intratumoral amyloid deposition or fibrosis, encapsulation, lymphatic invasion, vascular invasion, extrathyroidal extension (microscopic or gross), and resection margin status (Supplementary Fig. 1). Mitotic count was assessed in the most mitotically active area. Tumor necrosis was defined as zones or clusters of cells showing degenerating cytoplasm and punctate, karyorrhectic nuclear debris, regardless of extent. Infarct-type necrosis associated with a fibroblastic stromal reaction, hemorrhage, or identifiable fine-needle aspiration tract was disregarded. Nuclear pleomorphism was semi-quantitatively graded as mild, moderate, and marked. Based on the criteria used in a previous study [16], prominent nucleoli were defined as nucleoli visible at 100x magnification, similar to Fuhrman nuclear grade 3 in renal cell carcinoma. A sheet-like growth pattern was defined as areas composed of smaller cells with little cytoplasm and diffuse growth, superficially resembling small cell carcinoma but lacking its classic features such as nuclear molding and intense mitotic activity. The proportions of spindle cell component, sheet-like growth pattern, and amyloid deposition or fibrosis were estimated using all tumor sections. Lymphovascular invasion was defined using the criteria outlined in the 2022 WHO classification of thyroid tumors [27]. Following local review, two unstained slides from the most representative tumor block were sent to the central institution for H&E staining and Ki-67 immunohistochemistry. Two endocrine pathologists (M.K. and H.N.) centrally reviewed these slides, and any discrepancies with the initial local assessments were resolved by consensus.

### Ki-67 Immunohistochemistry and Digital Image Analysis

Ki-67 immunohistochemistry (clone MIB-1, Dako, Agilent Technologies, Santa Clara, CA, USA) was performed using the Ultraview Universal DAB Detection Kit in a BenchMark XT autostainer (Ventana Medical Systems, Tucson, AZ, USA). Immunostained slides were digitized using the Aperio GT 450 scanner (Leica Biosystems, Vista, CA, USA) at 40x magnification and analyzed using the Sectra digital pathology platform (Sectra AB, Linköping, Sweden).

Ki-67 indices were assessed by two pathologists (M.K. and H.N.) in a blinded manner, using two different methods. For the hotspot method, the area showing the highest proliferative activity was selected and a minimum of 1000 tumor cells were counted within that region. For the average method, the Ki-67 index was calculated across three representative tumor regions, with at least 1000 tumor cells counted in each. Ki-67 quantification was performed by DIA using a dedicated scoring application (Sectra AB). After a pathologist delineated a region of interest, the software automatically enumerated a number of positive cells within that area, with manual adjustment permitted if necessary. The pathologists verified that the cells quantified as positive were indeed tumor cells and that no true-positive tumor cells were omitted. Nuclear staining of any intensity (weak to strong) was considered positive (Supplementary Fig. 2). The IMTCGS grading was applied as follows: tumors meeting at least one of the criteria—mitotic count ≥ 5/2 mm^2^, Ki-67 index ≥ 5%, or the presence of tumor necrosis—were classified as high-grade, whereas all others were classified as low-grade [17]. Grading was additionally evaluated using alternative Ki-67 labeling index cutoffs, including 3% and the optimal cutoff derived from receiver operating characteristic (ROC) curve analysis for predicting recurrence-free survival.

### Statistical Analysis

All statistical analyses were performed using IBM SPSS Statistics for Windows, version 19.0 (IBM Corp., Armonk, NY, USA). Associations between Ki-67 index categories and clinicopathological features were evaluated using chi-square tests or Fisher’s exact tests, as appropriate. For these analyses, continuous variables, such as preoperative and postoperative CEA and calcitonin levels, were dichotomized into high and low groups based on their median values. Regarding histopathological features, the proportion of sheet-like growth pattern was categorized using a 10% cutoff, whereas the spindle cell component and amyloid deposition/fibrosis were divided at a 50% threshold in accordance with the previously published criteria [16]. In addition to the established 5% threshold, alternative cutoff values for the Ki-67 index were derived by ROC curve analysis for predicting recurrence based on the Youden index. Recurrence-free survival (RFS) was defined as the interval from primary surgery to the first documented local, regional, or distant recurrence. Survival analyses and ROC curve-based cutoff derivation were restricted to patients without distant metastasis at initial diagnosis. Survival curves were constructed using the Kaplan–Meier method and compared via the log-rank test. Univariate and multivariate analyses were conducted using Cox proportional hazards regression models. To avoid multicollinearity, correlations among variables significant in the univariate analysis were assessed using Spearman’s rank correlation; when two variables were highly correlated (|ρ| > 0.5), only the variable considered more clinically relevant was included in the multivariate model. Significant variables (*p* < 0.05) in univariate analysis were included in the multivariate analysis using a backward stepwise selection method. Hazard ratios (HRs) and 95% confidence intervals (CIs) were calculated for the significant variables. All *p* values were two-sided, and *p* values less than 0.05 were considered statistically significant.

## Results

### Characteristics of Study Population

The clinical characteristics of the 187 MTC patients included in this study are summarized in Table 1. The median patient age was 56 years (range, 14–84 years), with 108 female (57.8%) and 79 male (42.2%) patients. Pathogenic germline *RET* mutations were identified in 18 (23.1%) of the 78 patients who underwent germline testing. At the time of diagnosis, 8 patients (4.3%) presented with distant metastasis. Following surgical resection, 125 cases (66.8%) were classified as pT1 and 62 cases (33.2%) as pT2-T4. Regarding lymph node status, 31 (16.6%) and 49 (26.2%) cases were categorized as pN1a and pN1b, respectively. According to the AJCC staging system, 106 patients (56.7%) had stage I–II disease, whereas 81 (43.3%) had stage III–IV disease. Adjuvant radiotherapy and chemotherapy were administered to 13 (7.0%) and 6 (3.2%) patients, respectively.

**Table 1:** Clinical characteristics of the study population

Characteristics	Number (%)
Sex	
Male	79 (42.2%)
Female	108 (57.8%)
Age [median (range)] (years)	56 (14–84)
Preoperative CEA [median (range)] (ng/mL)	12.6 (0.5–1298.0)
Preoperative calcitonin [median (range)] (pg/mL)	214.0 (4.6-100000.0)
Postoperative CEA [median (range)] (ng/mL)	3.3 (0.1-402.2)
Postoperative calcitonin [median (range)] (pg/mL)	5.0 (0.0-4330.0)
*RET* germline mutation	
Absent	60 (32.1%)
Present	18 (9.6%)
Not assessed	109 (58.3%)
pT category	
pT1	125 (66.8%)
pT2-T4	62 (33.2%)
pN category	
pNx	7 (3.7%)
pN0	100 (53.5%)
pN1a	31 (16.6%)
pN1b	49 (26.2%)
M category	
M0	175 (93.6%)
M1	8 (4.3%)
Mx	4 (2.1%)
AJCC stage	
I-II	106 (56.7%)
III-IV	81 (43.3%)
Adjuvant radiation therapy	
Not done	173 (92.5%)
Done	13 (7%)
Unknown	1 (0.5%)
Adjuvant chemotherapy	
Not done	180 (96.3%)
Done	6 (3.2%)
Unknown	1 (0.5%)
Last FU status	
Alive with no disease	160 (85.6%)
Alive with disease	13 (7%)
Dead due to the disease	2 (1.1%)
Dead due to other causes	6 (3.2%)
FU loss	6 (3.2%)

FU, follow-up; AJCC, American Joint Committee on Cancer

As for histopathological features, the median tumor size was 1.4 cm (range, 0.3–6.9 cm), and 41 cases (21.9%) presented as multiple tumors. Most cases showed no or low mitotic activity: 108 cases (57.8%) had 0 mitoses/2 mm², 59 (31.6%) had 1–2/2mm^2^, and only 5 (2.7%) had ≥ 5/2mm^2^. Lymphatic invasion was present in 56 cases (29.9%) and vascular invasion in 19 cases (10.2%). Microscopic and gross extrathyroidal extension was found in 42 (22.5%) and 8 cases (4.3%), respectively. Other histopathological features of the tumors are summarized in Supplementary Table 1.

### Proportion of High Grade MTC

**Fig. 1:**
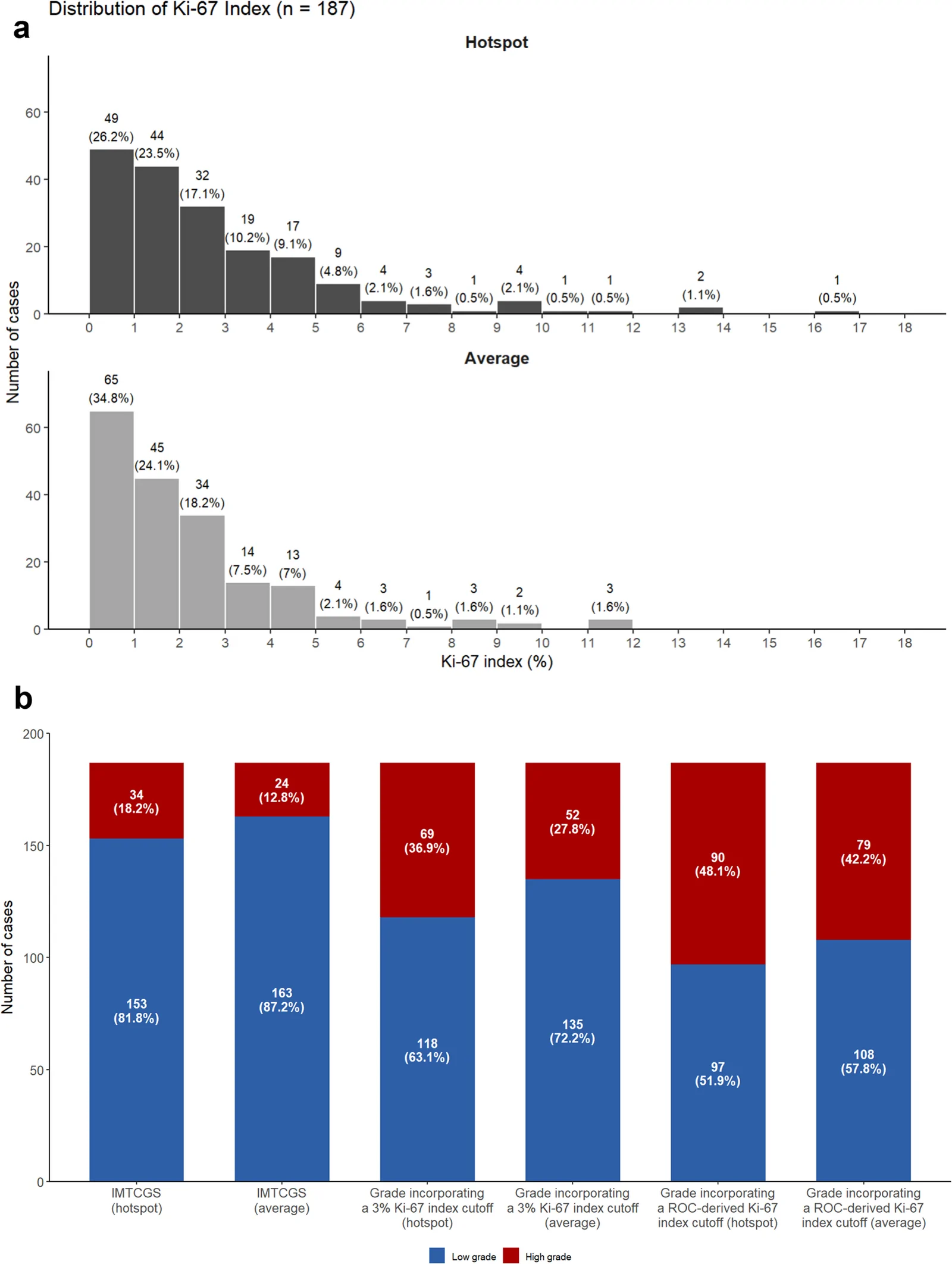
(**a**) Distribution of hotspot (upper) and average (lower) Ki-67 indices among the cases. (**b**) Distribution of grade according to various cutoffs. A total of 34 (18.2%) and 24 (12.8%) cases were classified as high-grade, using the IMTCGS by hotspot and average assessment methods, respectively. When a 3% cutoff was applied while maintaining the other two criteria, the numbers of high-grade cases were 69 (36.9%) with the hotspot method and 52 (27.8%) with the average method. Applying the ROC curve–derived cutoffs (2.25%, for hotspot; 1.85%, for average) rendered 90 (48.1%) and 79 (42.2%) high-grade cases, using the hotspot and average methods, respectively

The median Ki-67 indices assessed by hotspot and average methods were 2.1% (range, 0.1–17.0%) and 1.7% (range, 0–12.0%), respectively (Fig. 1A). Using the IMTCGS-proposed 5% Ki-67 index cutoff, 34 (18.2%) and 24 (12.8%) cases were classified as high-grade by hotspot and average assessment methods, respectively. When a 3% cutoff was applied while maintaining the other two criteria, the numbers of high-grade cases increased to 69 (36.9%) with the hotspot method and 52 (27.8%) with the average method. When the ROC curve–derived cutoffs were applied (2.25%, for hotspot; 1.85%, for average), 90 cases (48.1%) and 79 (42.2%) were categorized as high-grade using the hotspot and average methods, respectively (Fig. 1B).

### Relationship between High Ki-67 Index and Clinicopathological Characteristics

Using the 5% cutoff of the IMTCGS, a high Ki-67 index was significantly associated with several adverse clinical features, regardless of whether it was assessed using the hotspot or average method (Table 2). A high Ki-67 index assessed by both methods was commonly associated with a high pT category (pT2-T4), lateral node metastasis, distant metastasis at diagnosis, and advanced AJCC stage (all *p* < 0.05). Patients with MTCs showing a high Ki-67 index were also more likely to receive adjuvant radiation therapy and chemotherapy (all *p* < 0.05). Additionally, a high Ki-67 index evaluated by the hotspot method was associated with higher postoperative serum calcitonin levels and *RET* germline mutation (all *p* < 0.05). As for histopathological features, a high Ki-67 index was significantly associated with large tumor size (≥ 2 cm), increased mitotic activity (≥ 3 mitoses/2 mm²), tumor necrosis, lymphovascular invasion, and positive resection margin status, irrespective of assessment methods (all *p* < 0.05; Table 3). Similar associations were observed for different cutoffs: 3% (hotspot or average method), 2.25% (ROC curve-derived, hotspot), and 1.85% (ROC curve-derived, average) (Supplementary Table 2).

**Table 2:** Relationship between high Ki-67 index (≥ 5%) assessed by hotspot and average methods and clinical characteristics of the patients

Characteristics	Ki-67 index (Hotspot)			Ki-67 index (Average)		
	< 5% (*n* = 161)	≥ 5% (*n* = 26)	*p*-value	< 5% (*n* = 171)	≥ 5% (*n* = 16)	*p*-value
Sex			0.664			0.899
Male	67 (41.6%)	12 (46.2%)		72 (41.2%)	7 (43.8%)	
Female	94 (58.4%)	14 (53.8%)		99 (57.8%)	9 (56.3%)	
Age (years)			0.996			0.533
<50	62 (38.5%)	10 (38.5%)		67 (39.2%)	5 (31.3%)	
≥50	99 (61.5%)	16 (61.5%)		104 (60.8%)	11 (68.8%)	
Preoperative CEA (*n* = 112)			0.074			0.113
Low	53 (53.5%)	3 (23.1%)		55 (52.4%)	1 (14.3%)	
High	46 (46.5%)	10 (76.9%)		50 (47.6%)	6 (85.7%)	
Preoperative calcitonin (*n* = 171)			0.084			0.431
Low	77 (52.4%)	8 (33.3%)		79 (50.6%)	6 (40.0%)	
High	70 (47.6%)	16 (66.7%)		77 (49.4%)	9 (60.0%)	
Postoperative CEA (*n* = 155)			0.375			0.368
Low	68 (51.1%)	9 (40.9%)		73 (51.0%)	4 (33.3%)	
High	65 (48.9%)	13 (59.1%)		70 (48.9%)	8 (66.7%)	
Postoperative calcitonin (*n* = 181)			0.029			0.107
Low	68 (43.6%)	5 (20.0%)		70 (42.2%)	3 (20.0%)	
High	88 (56.4%)	20 (80.0%)		96 (57.8%)	12 (80.0%)	
*RET* germline mutation (*n* = 78)			0.031			0.343
Absent	53 (81.5%)	7 (53.8%)		56 (78.9%)	4 (57.1%)	
Present	12 (18.5%)	6 (46.2%)		15 (21.1%)	3 (42.9%)	
pT category			0.004			< 0.001
pT1	114 (70.8%)	11 (42.3%)		121 (70.8%)	4 (25.0%)	
pT2-4	47 (29.2%)	15 (57.7%)		50 (29.2%)	12 (75.0%)	
pN category (*n* = 180)			< 0.001			< 0.001
pN0-1a	121 (78.1%)	10 (40.0%)		127 (77.4%)	4 (25.0%)	
pN1b	34 (21.9%)	15 (60.0%)		37 (22.6%)	12 (75.0%)	
M category (*n* = 183)			0.001			0.002
M0	155 (98.1%)	20 (80.0%)		164 (97.6%)	11 (73.3%)	
M1	3 (1.9%)	5 (20.0%)		4 (2.4%)	4 (26.7%)	
AJCC			0.004			0.015
I-II	98 (60.9%)	8 (30.8%)		102 (59.6%)	4 (25.0%)	
III-IV	63 (39.1%)	18 (69.2%)		69 (40.4%)	12 (75.0%)	
Adjuvant radiation therapy (*n* = 186)			0.021			< 0.001
Not done	152 (95.0%)	21 (80.8%)		162 (95.3%)	11 (68.8%)	
Done	8 (5.0%)	5 (19.2%)		8 (4.7%)	5 (31.3%)	
Adjuvant chemotherapy (*n* = 186)			0.004			0.009
Not done	158 (98.8%)	22 (84.6%)		167 (98.2%)	13 (81.3%)	
Done	2 (1.2%)	4 (15.4%)		3 (1.8%)	3 (18.8%)	

AJCC, American Joint Committee on Cancer

**Table 3:** Relationship between high Ki-67 index (≥ 5%) assessed by hotspot and average methods and histopathological features of tumors

Histopathological features	Ki-67 index (Hotspot)			Ki-67 index (Average)		
	< 5% (*n* = 161)	≥ 5% (*n* = 26)	*p*-value	< 5% (*n* = 171)	≥ 5% (*n* = 16)	*p*-value
Tumor size			0.008			< 0.001
<2 cm	116 (72.0%)	12 (46.2%)		124 (72.5%)	4 (25.0%)	
≥2 cm	45 (28.0%)	14 (53.8%)		47 (27.5%)	12 (75.0%)	
Multiplicity			0.507			0.346
Single	127 (78.9%)	19 (73.1%)		135 (78.9%)	11 (68.8%)	
Multiple	34 (21.1%)	7 (26.9%)		36 (21.1%)	5 (31.3%)	
Mitotic count (/2 mm^2^)			0.143			0.059
<5	158 (98.1%)	24 (92.3%)		168 (98.2%)	14 (87.5%)	
≥5	3 (1.9%)	2 (7.7%)		3 (1.8%)	2 (12.5%)	
Mitotic count (/2 mm^2^)			< 0.001			0.017
<3	149 (92.5%)	18 (69.2%)		156 (91.2%)	11 (68.8%)	
≥3	12 (7.5%)	8 (30.8%)		15 (8.8%)	5 (31.3%)	
Tumor necrosis			0.001			< 0.001
Absent	156 (96.9%)	20 (76.9%)		166 (97.1%)	10 (62.5%)	
Present	5 (3.1%)	6 (23.1%)		5 (2.9%)	6 (37.5%)	
Spindle cell component			0.572			0.281
<50%	137 (85.1%)	21 (80.8%)		146 (85.4%)	12 (75%)	
≥50%	24 (14.9%)	5 (19.2%)		25 (14.6%)	4 (25%)	
Sheet-like pattern			0.751			0.131
<10%	141 (87.6%)	22 (84.6%)		151 (88.3%)	12 (75%)	
≥10%	20 (12.4%)	4 (15.4%)		20 (11.7%)	4 (25%)	
Amyloid deposition/fibrosis			0.089			0.506
<50%	134 (83.2%)	18 (69.2%)		140 (81.9%)	12 (75%)	
≥50%	27 (16.8%)	8 (30.8%)		31 (18.1%)	4 (25%)	
Encapsulation			0.075			0.482
Encapsulated	98 (60.9%)	11 (42.3%)		101 (59.1%)	8 (50%)	
Not encapsulated	63 (39.1%)	15 (57.7%)		70 (40.9%)	8 (50%)	
Lymphovascular invasion			0.002			0.005
Absent	118 (73.3%)	11 (42.3%)		123 (71.9%)	6 (37.5%)	
Present	43 (26.7%)	15 (57.7%)		48 (28.1%)	10 (62.5%)	
Gross extrathyroidal extension			0.308			0.142
Absent	155 (96.3%)	24 (92.3%)		165 (96.5%)	14 (87.5%)	
Present	6 (3.7%)	2 (7.7%)		6 (3.5%)	2 (12.5%)	
Resection margin status			0.002			0.022
Negative	158 (98.1%)	21 (80.8%)		166 (97.1%)	13 (81.3%)	
Positive	3 (1.9%)	5 (19.2%)		5 (2.9%)	3 (18.8%)	

AJCC, American Joint Committee on Cancer

### Prognostic Significance of Histologic Grade and Ki-67 Index

During a median follow-up of 83 months (range, 1–123 months), disease recurrence occurred in 15 (8.6%) of 175 patients who initially presented with localized disease without distant metastasis. At the last follow-up of all 187 patients, 160 patients (85.6%) were alive with no evidence of disease, and 2 patients (1.1%) had died of MTC.

**Fig. 2:**
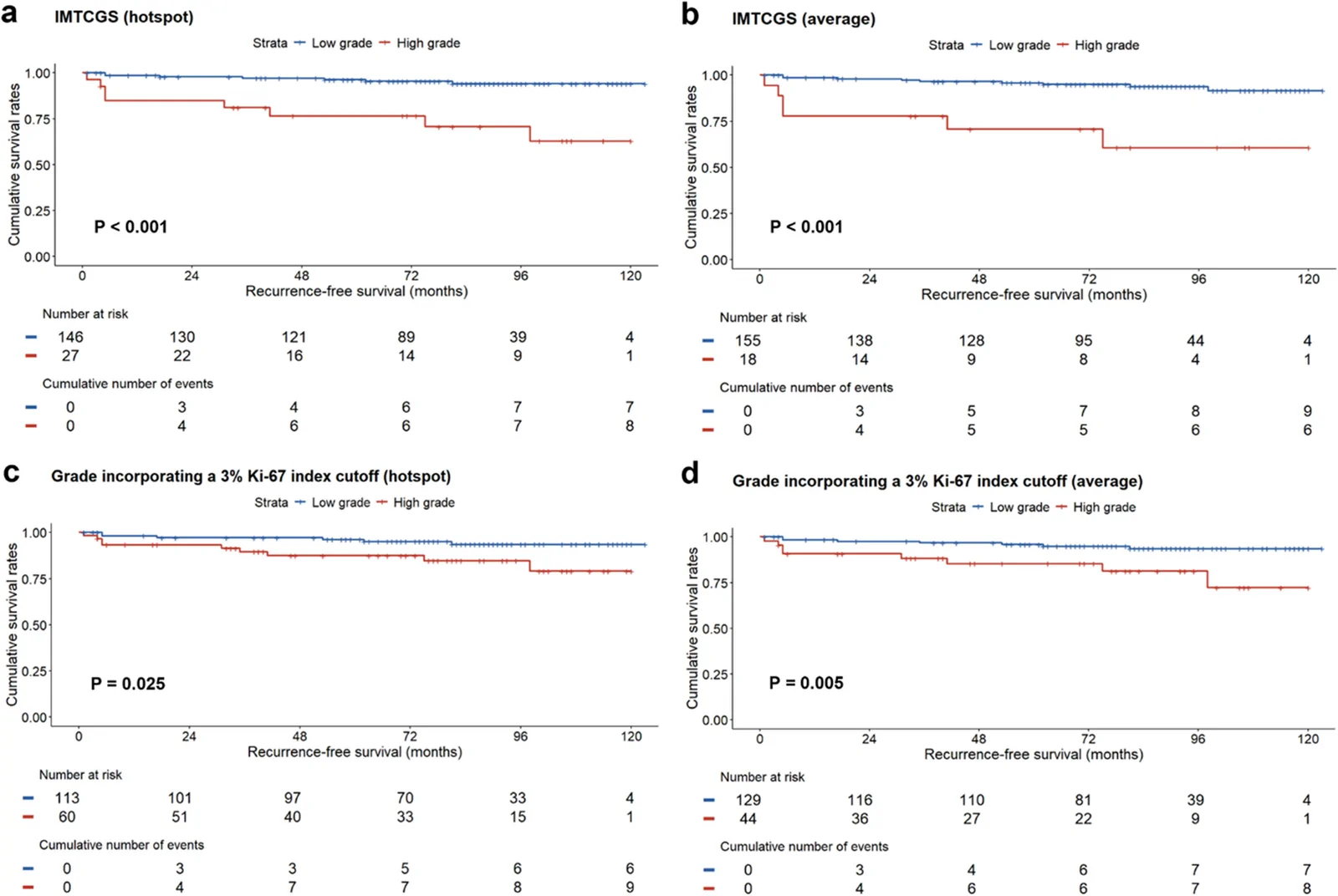
Kaplan–Meier survival analysis for recurrence-free survival (RFS). Medullary thyroid carcinomas (MTCs) classified as high-grade by the IMTCGS, using either (**a**) the hotspot or (**b**) the average method for Ki-67 assessment, were significantly associated with shorter RFS (both *p* < 0.05). High-grade tumors defined by a 3% Ki-67 cutoff, using either (**c**) the hotspot or (**d**) the average method, were also significantly associated with shorter RFS (both *p* < 0.05)

Kaplan–Meier survival analysis demonstrated significantly shorter RFS in patients with high-grade MTC determined by the IMTCGS using the hotspot (*p* < 0.001, by log-rank test) and average (*p* < 0.001, by log-rank test) methods (Fig. 2A-B). High-grade tumors incorporating a 3% Ki-67 index cutoff either by the hotspot (*p* = 0.025, by log-rank test) or average (*p* = 0.005, by log-rank test) method were also significantly associated with shorter RFS (Fig. 2C-D).

In univariate analyses using the Cox proportional hazards regression model (Supplementary Table 3), the individual grading parameters, a Ki-67 index ≥ 5% assessed by either the hotspot or average method, mitotic count ≥ 5/2 mm², and tumor necrosis were associated with inferior RFS (all *p* < 0.05). However, alternative cutoffs, including 3% and the ROC curve–derived thresholds, evaluated by either the hotspot or average method, failed to demonstrate statistically significant associations with RFS, although higher Ki-67 indices showed trends toward shorter RFS. Among other clinical and histopathological characteristics, male sex, preoperative CEA level, postoperative CEA level, postoperative calcitonin level, multiplicity, amyloid deposition and fibrosis, lack of encapsulation, lymphovascular invasion, gross extrathyroidal extension, pT2-4, pN1b, and AJCC III-IV were each significantly associated with inferior RFS (all *p* < 0.05).

### Multivariate Analysis for RFS using Histologic Grade and its Individual Components

As a next step, multivariate Cox proportional hazards regression analyses for RFS were performed, incorporating histologic grade either as a composite variable or through its individual parameters (tumor necrosis, mitosis, and Ki-67 index) in separate models. To address potential multicollinearity among highly correlated clinicopathological variables associated with RFS (Supplementary Table 4)—such as preoperative vs. postoperative CEA levels (ρ = 0.820), preoperative CEA vs. postoperative calcitonin levels (ρ = 0.622), postoperative CEA vs. postoperative calcitonin levels (ρ = 0.520), tumor size vs. pT category (ρ = 0.917), and pN1b vs. AJCC stage III-IV (ρ = 0.651)—postoperative calcitonin levels, pT category, and pN1b were selected for inclusion in the final multivariate models.

The IMTCGS high-grade classification using the hotspot Ki-67 index was an independent predictor of shorter RFS on multivariate analysis (HR, 3.217; 95% CI, 1.080–9.587; *p* = 0.036), along with male sex (HR, 4.379; 95% CI, 1.206–15.898; *p* = 0.025), postoperative serum calcitonin levels (HR, 1.002; 95% CI, 1.000–1.003; *p* = 0.021), and pN1b (HR, 31.478; 95% CI, 3.657–270.953; *p* = 0.002) (Table 4). However, the IMTCGS high-grade classification based on the average Ki-67 index was not an independent prognostic factor on multivariate analysis. Male sex (HR, 3.560; 95% CI, 1.028–12.388; *p* = 0.045), postoperative serum calcitonin levels (HR, 1.002; 95% CI, 1.000–1.003; *p* = 0.013) and pN1b (HR, 42.077; 95% CI, 5.131–345.059; *p* < 0.001) remained as independent predictors of inferior RFS (Table 4). When tumor necrosis, mitotic count (≥ 5/2 mm²), and Ki-67 index (≥ 5%, assessed by either hotspot or average) were evaluated as individual covariates in separate models, none of these constituent components or the other clinicopathological variables retained independent prognostic significance for RFS.

**Table 4:** Multivariate Cox regression analysis for recurrence-free survival based on hotspot or average Ki-67 index using a 5% cutoff

Parameters	Comparison	HR	95% CI	*p*-value
Sex	Female vs. Male	4.379	1.206–15.898	0.025
Postoperative calcitonin	continuous	1.002	1.000–1.003	0.021
pT2-4	pT1 vs. pT2-4	-	-	-
pN1b	pN0-1a vs. pN1b	31.478	3.657–270.953	0.002
IMTCGS (hotspot)	Low grade vs. High grade	3.217	1.080–9.587	0.036
Multiplicity	Single vs. Multiple	-	-	-
Encapsulation	Present vs. Absent	-	-	-
Amyloid deposition/fibrosis	< 50% vs. ≥50%	-	-	-
Lymphovascular invasion	Absent vs. Present	-	-	-
Extrathyroidal extension (gross)	Absent vs. present	-	-	-
Sex	Female vs. Male	3.569	1.028–12.388	0.045
Postoperative calcitonin	continuous	1.002	1.000–1.003	0.013
pT2-4	pT1 vs. pT2-4	-	-	-
pN1b	pN0-1a vs. pN1b	42.077	5.131–345.059	< 0.001
IMTCGS (average)	Low grade vs. High grade	-	-	-
Multiplicity (multiple)	Single vs. Multiple	-	-	-
Encapsulation	Present vs. Absent	-	-	-
Amyloid deposition/fibrosis	< 50% vs. ≥50%	-	-	-
Lymphovascular invasion	Absent vs. Present	-	-	-
Extrathyroidal extension (gross)	Absent vs. present	-	-	-

*HR* hazard ratio, *CI* confidence interval, *IMTCGS* International Medullary Thyroid Carcinoma Grading System

In multivariate analyses incorporating a 3% Ki-67 index cutoff as a high-grade criterion, histologic grading failed to retain independent prognostic significance, irrespective of the Ki-67 assessment method employed (Supplementary Table 5).

## Discussion

In this multicenter study of 187 MTCs, we validated the prognostic utility of the IMTCGS and addressed a key unresolved methodological issue within the grading framework: the optimal approach for Ki-67 assessment. Using DIA, we demonstrated that high-grade status according to the hotspot-based IMTCGS remained independently associated with RFS after multivariable adjustment, whereas average-based grading did not. This suggests that the proliferative index evaluated specifically in the hotspot carries significant prognostic value in MTCs. To our knowledge, this is the first study to systematically compare hotspot- and average-based Ki-67 assessment methods within the IMTCGS framework using DIA.

Our findings confirmed the prognostic relevance of the IMTCGS in a Korean cohort. High-grade tumors, defined by the presence of at least one of the following features—mitotic count ≥ 5/2 mm², Ki-67 index ≥ 5%, or tumor necrosis—were significantly associated with shorter RFS. Notably, the IMTCGS grading based on hotspot Ki-67 assessment served as an independent prognostic factor for RFS, whereas the average-based grading did not. Since the IMTCGS was proposed by Xu et al. [17], several studies mainly from Western countries [18, 20–22], with additional studies from China [23] and India [24] have confirmed its prognostic significance and clinical utility. The present study is the first to validate the clinical significance of the IMTCGS in a Korean cohort, further supporting its broader applicability across diverse patient populations.

A major finding of this study is that the prognostic performance of the IMTCGS is influenced by how the Ki-67 index is assessed. Although multiple studies have validated the IMTCGS using hotspot-based assessment [18–21, 23, 24], none has directly compared the clinical utility of hotspot- and average-based assessment approaches within the grading system. In our cohort, both hotspot and average Ki-67 measurements were associated with adverse clinicopathologic features and shorter RFS; however, only hotspot-based IMTCGS grading retained independent prognostic significance. Furthermore, a higher number of tumors were classified as high-grade when using hotspot assessment, compared with average assessment (18.2% vs. 12.8%). These findings suggest that average Ki-67 indices calculated from three representative tumor regions may dilute focal areas of high proliferative activity that are more relevant to biological behavior and clinical outcome. Although the Sydney study [16] and the study performed by Najdawi et al. [21], have suggested that Ki-67 labeling in MTC is often relatively homogeneous and may not require hotspot-based assessment, our findings indicate that the method of Ki-67 evaluation can meaningfully influence prognostic stratification. This observation is biologically plausible, as tumor behavior is likely driven by the most proliferative subclones rather than the average proliferative activity of the entire tumor. In addition, postoperative calcitonin level was associated with the hotspot but not with the average Ki-67 index at the 5% cutoff, further suggesting that hotspot assessment better reflect biologically aggressive disease. Although whole-slide analysis has been reported to provide more accurate prognostic stratification in gastroenteropancreatic NENs [28], we compared methods that are readily applicable in routine practice to provide practical guidance for MTC.

We also evaluated whether alternative Ki-67 thresholds (3% and ROC curve-derived cutoffs) might improve prognostic stratification compared with the IMTCGS-defined 5% cutoff. Xu et al. have demonstrated some degree of prognostic associations in alternative grading schemes, including the Sydney system [17]. However, intermediate-grade MTCs generally lacked prognostic distinction from low- and high-grade MTCs, contributing to the consensus toward the two-tiered IMTCGS [17]. Vissio et al. have also compared the prognostic significance of the IMTCGS and the Sydney system and found that the IMTCGS was an independent prognostic factor for disease-free survival whereas the Sydney system was not [18]. Accordingly, we evaluated various two-tiered cutoffs. Although the 3% hotspot and average cutoffs remained significant in univariate analysis when incorporated into modified IMTCGS grading models, this association was no longer significant after multivariable adjustment. Similarly, ROC curve-derived cutoffs failed to demonstrate prognostic performance. These findings indicate that neither the 3% cutoff in the Sydney grading system [16] nor the ROC curve-derived thresholds provide incremental prognostic value, supporting the clinical validity of the established 5% Ki-67 index cutoff within the IMTCGS framework. An additional strength of this study is the application of DIA for Ki-67 quantification. Although manual counting remains the gold standard for evaluating the Ki-67 index, it is labor-intensive and subject to interobserver variability. Previous studies performed in breast cancer [29, 30], and gastroenteropancreatic NEN [31, 32], have demonstrated good concordance between manual and digital Ki-67 assessment. Similarly, recent studies performed in MTCs have also demonstrated good agreement between the two methods, leaving only a few cases with discrepant grades, suggesting that DIA can provide reliable quantification of Ki-67 proliferative activity [33, 34]. Building on these observations, our study applied DIA in a large multicenter cohort and directly compared hotspot-based and average-based assessment strategies, which revealed the robust prognostic significance of the Ki-67 index. Our findings suggest that DIA-based quantification within pathologist-selected regions of interest provides a standardized and reproducible approach, supporting the integration of DIA-based hotspot Ki-67 assessment into routine clinical practice.

Notably, while the composite high-grade classification remained an independent predictor of RFS in multivariate analysis, none of its constituent components — tumor necrosis, mitotic count, or Ki-67 index — retained independent prognostic significance when evaluated separately. Part of this discrepancy likely reflects limited statistical power, as each individual criterion was met by only a small number of tumors. However, the distribution of high-grade cases in our cohort suggests that this finding also reflects the design of the IMTCGS itself: of 34 high-grade tumors by the hotspot method, 27 (79.4%) fulfilled only one criterion, 6 (17.6%) fulfilled two, and 1 (2.9%) fulfilled all three. Thus, each parameter alone identified only a subset of aggressive tumors, whereas the composite scheme, which assigns high-grade when any single criterion is met, captured a larger and biologically heterogeneous group of aggressive tumors. A similar pattern was reported by Fuchs et al. [16], where the combined grade outperformed its individual components. Although the small number of recurrences (*n* = 15) precluded subgroup analysis, the enrichment of recurrences and distant metastases among high-grade tumors supports the prognostic validity of the composite grading scheme. We also evaluated the prognostic significance of various clinicopathological parameters beyond mitotic count, tumor necrosis, and the Ki-67 index. Alongside the IMTCGS grade, male sex, postoperative calcitonin level, and lateral lymph node metastasis emerged as independent prognostic factors for RFS. These findings are generally in line with previous studies. Male sex has been associated with poor clinical outcomes in a number of studies [15, 17, 22, 35, 36]. Postoperative calcitonin level [17, 23, 37, 38], and lateral lymph node status [38, 39] have likewise been reported as independent prognostic factors.

Among other histologic features, amyloid deposition or fibrosis was associated with worse RFS on univariate analysis, whereas other histologic features, such as nuclear pleomorphism, prominent nucleoli, spindle cell morphology, sheet-like pattern, or resection margin status, were not. Similarly, spindle cell morphology and sheet-like pattern have not been established as independent prognostic factors in previous studies [16, 40], and conflicting results have been reported regarding nuclear pleomorphism, amyloid or fibrosis, or resection margin [15–17, 22, 35, 41, 42]. These inconsistencies may be attributable to the interobserver and interstudy variability inherent in the assessment of these histologic features. Indeed, this limitation motivated the development of grading systems based on more objective and quantifiable parameters, namely mitotic count, tumor necrosis, and the Ki-67 proliferative index.

This study has several limitations. First, its retrospective design may have introduced selection bias. Second, although Ki-67 immunohistochemistry and DIA were performed centrally, differences in tissue fixation and specimen processing among participating institutions may have affected Ki-67 measurements. Third, because disease-specific deaths were few (*n* = 2), survival analyses for disease-specific mortality could not be performed, highlighting the need for further studies with extended follow-up. Finally, the relative prognostic utility of hotspot- and average-based Ki-67 assessment requires validation in larger, independent cohorts.

In conclusion, this study validated the IMTCGS in a large Korean multicenter cohort and demonstrated that hotspot-based DIA of the Ki-67 index provides more robust prognostic stratification than average-based assessment within the IMTCGS framework. We also confirmed the prognostic relevance of the 5% Ki-67 cutoff and the applicability of DIA in grading MTC. These findings support the use of the IMTCGS in risk assessment of MTC and provide practical guidance for its implementation in routine practice.

## Supplementary Information

Below is the link to the electronic supplementary material.

Supplementary Material 1 (PDF 1.26 MB)

## Data Availability

The datasets used and/or analyzed during the current study are available from the corresponding author on reasonable request.
