# Supplementary material for: Validation of the International Medullary Thyroid Carcinoma Grading System in a Multicenter Cohort: Digital Image Analysis-Based Evaluation of Ki-67 Cutoffs and Assessment Methods
Source: Endocr Pathol. 2026 Nov 11;37(1):44. doi: 10.1007/s12022-026-09940-0 (PMC13754965; doi:10.1007/s12022-026-09940-0)
Supplement: Supplementary file 1 [file 12022_2026_9940_MOESM1_ESM.pdf]

Validation of the International Medullary Thyroid Carcinoma Grading System in a Multicenter Cohort: Digital Image Analysis-Based Evaluation of Ki-67 Cutoffs and Assessment Methods

Endocrine Pathology

Milim Kim[1]*, Hee Young Na[2*], Su-Jin Shin[3], Ji-Young Park[4], Chan Kwon Jung[5], Jang-Hee Kim[6], Soo Jin Jung[7], Jae Kyung Won[8], Hyeongchan Shin[9], Jin-Man Kim[10,11], Hee Jung Kwon[12], Jee Yeon Kim[13], Yeseul Kim[14], Ju Yeon Pyo[15], In Ho Choi[16], Lucia Kim[17], So Yeon Park[2]

[2]Department of Pathology, Seoul National University Bundang Hospital, Seoul National University College of Medicine, Seongnam, Republic of Korea

***Equally contributed**

Corresponding Author:

So Yeon Park

Department of Pathology, Seoul National University Bundang Hospital, Seoul National University College of Medicine, 82, Gumi-ro 173 Beon-gil, Bundang-gu, Seongnam, Gyeonggi 13620, Republic of Korea

TEL: +82-31-787-7712; FAX: +82-31-787-4012; E-mail: sypmd@snu.ac.kr

Supplementary Table S1. Summary of histopatholgical features of tumors

Histopathological features	Number (%)
Tumor size [median (range)] (cm)	1.4 (0.3-6.9)
Multiplicity	
Single	146 (78.1%)
Multiple	41 (21.9%)
Mitotic count (/2mm^2)	
0	108 (57.8%)
1 – 2	59 (31.6%)
3 – 4	15 (8%)
5 - 9	4 (2.1%)
10 - 13	1 (0.5%)
Atypical mitosis	
Absent	173 (92.5%)
Present	14 (7.5%)
Tumor necrosis	
Absent	176 (94.1%)
Present	11 (5.9%)
Nuclear pleomorphism	
Mild to moderate	158 (84.5%)
Marked	29 (15.5%)
Prominent nucleoli	
Absent	109 (58.3%)
Present	78 (41.7%)
Spindle cell component [median (range)] (%)	0 (0-100)
Sheet-like pattern [median (range)] (%)	0 (0-60)
Amyloid deposition/fibrosis [median (range)] (%)	20 (0-80)
Encapsulation	
Partially or totally encapsulated	109 (58.3%)
Not encapsulated	78 (41.7%)
Lymphatic invasion	
Absent	131 (70.1%)
Present	56 (29.9%)
Vascular invasion	
Absent	168 (89.8%)
Present	19 (10.2%)
Extrathyroidal extension	
Absent	137 (73.3%)
Microscopic	42 (22.5%)
Gross	8 (4.3%)
Resection margin	
Negative	179 (95.7%)
Positive	8 (4.3%)

Supplementary Table S2. Association between clinicopathological characteristics and high Ki-67 index defined by alternative cutoffs (3% and ROC-derived) using hotspot and average methods

Clinicopatholgical characteristics	High Ki-67 index (≥3%)		High Ki-67 index (ROC-derived)	
	Hotspot method p-value	Average method p-value	Hotspot method p-value	Average method p-value
Preoperative CEA (high)	0.033	0.01	0.008	0.008
Preoperative calcitonin (high)	0.014	NS	0.003	0.007
Postoperative CEA (high)	NS	NS	0.044	0.042
Postoperative calcitonin (high)	NS	NS	NS	NS
pT2-4	<0.001	0.001	<0.001	<0.001
pN1b	0.004	0.013	0.014	0.002
M1	<0.001	0.005	NS	0.001
AJCC III-IV	0.018	NS	NS	NS
Adjuvant radiation therapy	NS	NS	0.009	NS
Adjuvant chemotherapy	0.017	0.003	0.024	0.007
Tumor size ≥ 2cm	<0.001	0.001	<0.001	<0.001
Mitotic count ≥ 5 (/2mm^2)	NS	NS	NS	0.017
Mitotic count ≥ 3 (/2mm^2)	0.002	<0.001	0.002	<0.001
Tumor necrosis	0.001	0.018	0.025	0.013
Lymphovascular invasion	0.001	0.049	0.001	0.007
Extrathyroidal extension (gross)	NS	NS	NS	NS
Positive resection margin	0.017	0.016	<0.001	0.023

ROC, receiver operating characteristic; AJCC, American Joint Committee on Cancer

Supplementary Table 3. Univariate Cox regression analysis for recurrence-free survival

Category	Parameters	Comparison	HR	95% CI	p-value
Clinical characteristics	Sex	Female vs. Male	4.044	1.286 – 12.714	0.017
	Age	Continuous	0.987	0.956 - 1.020	0.435
	Preoperative CEA	Continuous	1.002	1.000 – 1.005	0.026
	Preoperative calcitonin	Continuous	1.000	1.000 – 1.000	0.913
	Postoperative CEA	Continuous	1.010	1.006 – 1.015	<0.001
	Postoperative calcitonin	Continuous	1.003	1.002 - 1.005	<0.001
	RET germline mutation	Absent vs. Present	2.470	0.588 – 10.375	0.217
	pT category	pT1 vs. pT2-4	5.324	1.815 – 15.617	0.002
	pN category	pN0-N1a vs. pN1b	56.535	7.373 – 433.478	<0.001
Histopathological features	Tumor size	<2cm vs. ≥2cm	3.137	1.135 - 8.667	0.027
	Multiplicity	Absent vs. Present	4.444	1.610 - 12.261	0.004
	Mitotic count (/2mm^2)	<5 vs. ≥5	12.294	2.739 – 55.176	0.001
	Tumor necrosis	Absent vs. Present	8.293	2.265 - 30.365	0.001
	Nuclear pleomorphism	Mild to moderate vs. Marked	1.521	0.429 – 5.396	0.516
	Prominent nucleoli	Absent vs. Present	1.550	0.562 – 4.276	0.397
	Spine cell component	<50% vs. ≥50%	0.987	0.223 – 4.379	0.986
	Sheet-like pattern	<10% vs. ≥10%	1.791	0.503 – 6.374	0.369
	Amyloid deposition/Fibrosis	<50% vs. ≥50%	4.163	1.502 – 11.540	0.006
	Encapsulation	Present vs. Absent	3.412	1.164 - 10.005	0.025
	Lymphovascular invasion	Absent vs. Present	6.125	2.088 - 17.966	0.001
	Gross extrathyroidal extension	Absent vs. Present	14.313	3.923 – 52.215	<0.001
	Resection margin	Not involved vs. Involved	3.612	0.471 – 27.705	0.217
Ki-67 index	5% cutoff (hotspot)	<5% vs. ≥5%	5.155	1.829 – 14.528	0.002
	5% cutoff (average)	<5% vs. ≥5%	5.861	1.861 – 18.454	0.003
	3% cutoff (hotspot)	<3% vs. ≥3%	1.929	0.699 – 5.323	0.205
	3% cutoff (average)	<3% vs. ≥3%	2.662	0.946 – 7.490	0.064
	ROC-derived cutoff (hotspot)	<2.25% vs. ≥2.25%	2.053	0.730 – 5.772	0.173
	ROC-derived cutoff (average)	<1.85% vs. ≥1.85%	2.183	0.776 – 6.135	0.139
Grading system	IMTCGS (hotspot)	Low grade vs. High grade	6.925	2.505 – 19.146	<0.001
	IMTCGS (average)	Low grade vs. High grade	7.150	2.534 – 20.173	<0.001
	Grading incorporating a 3% Ki-67 index cutoff (hotspot)	Low grade vs. High grade	3.073	1.093 – 8.643	0.033
	Grading incorporating a 3% Ki-67 index cutoff (average)	Low grade vs. High grade	3.980	1.407 – 10.752	0.009
	Grading incorporating a ROC-derived Ki-67 cutoff (hotspot)	Low grade vs. High grade	1.931	0.687 – 5.428	0.212
	Grading incorporating a ROC-derived Ki-67 cutoff (average)	Low grade vs. High grade	2.121	0.754 – 5.965	0.154

HR, hazard ratio; CI, confidence interval; IMTCGS, International Medullary Thyroid Carcinoma Grading System

Supplementary Table S4. Correlations between clinicopathological variables associated with recurrence-free survival of the patients

Variables	Sex	Preoperative CEA	Postoperative CEA	Postoperative calcitonin	pT category	pN category	Tumor size	Multiplicity	Mitotic count	Tumor necrosis	Encapsulation	Amyloid/ fibrosis	LVI	Gross ETE	AJCC III-IV
Sex	-	-0.026 (0.796)	0.159 (0.057)	0.124 (0.109)	0.059 (0.442)	0.129 (0.096)	0.004 (0.962)	0.088 (0.244)	0.026 (0.736)	0.182 (0.016)	0.076 (0.316)	0.093 (0.220)	0.155 (0.040)	0.103 (0.174)	0.161 (0.033)
Preoperative CEA		-	**0.820 (<0.001)**	**0.622 (<0.001)**	**0.573 (<0.001)**	0.464 (<0.001)	**0.516 (<0.001)**	0.236 (0.017)	0.093 (0.353)	0.147 (0.140)	-0.111 (0.267)	0.119 (0.232)	0.310 (0.001)	0.168 (0.091)	0.474 (<0.001)
Postoperative CEA			-	**0.510 (<0.001)**	0.403 (<0.001)	0.413 (<0.001)	0.405 (<0.001)	0.211 (0.011)	0.179 (0.033)	0.178 (0.033)	-0.125 (0.138)	0.053 (0.527)	0.213 (0.011)	0.066 (0.435)	0.311 (<0.001)
Postoperative calcitonin				-	0.468 (<0.001)	0.464 (<0.001)	0.438 (<0.001)	0.255 (0.001)	0.096 (0.324)	0.157 (0.041)	0.028 (0.720)	0.063 (0.418)	0.463 (<0.001)	0.179 (0.020)	**0.538 (<0.001)**
pT category					-	0.320 (<0.001)	**0.917 (<0.001)**	0.173 (0.022)	0.152 (0.045)	0.186 (0.014)	-0.192 (0.011)	0.026 (0.734)	0.217 (0.004)	0.235 (0.002)	0.209 (0.005)
pN category						-	0.223 (0.004)	0.410 (<0.001)	0.139 (0.073)	0.238 (0.002)	0.157 (0.042)	0.211 (0.006)	0.401 (<0.001)	0.284 (<0.001)	**0.651 (<0.001)**
Tumor size							-	0.096 (0.204)	0.157 (0.038)	0.194 (0.010)	-0.277 (<0.001)	-0.062 (0.419)	0.178 (0.018)	-0.012 (0.874)	0.155 (0.041)
Multiplicity								-	0.105 (0.167)	0.034 (0.656)	0.171 (0.024)	0.046 (0.545)	0.235 (0.002)	0.290 (<0.001)	0.334 (<0.001)
Mitotic count									-	-0.031 (0.682)	-0.045 (0.553)	-0.071 (0.351)	0.163 (0.031)	-0.023 (0.759)	0.031 (0.682)
Tumor necrosis										-	-0.045 (0.551)	0.211 (0.005)	0.267 (<0.001)	0.164 (0.030)	0.190 (0.012)
Encapsulation											-	0.146 (0.053)	0.238 (0.002)	0.190 (0.012)	0.177 (0.019)
Amyloid/fibrosis												-	0.184 (0.015)	0.229 (0.002)	0.263 (<0.001)
LVI													-	0.163 (0.031)	**0.518 (<0.001)**
Gross ETE														-	0.187 (0.013)
AJCC III-IV															-

LVI, lymphovascular invasion; ETE, extrathyroidal extension; AJCC, American Joint Committee on Cancer; pT category, pT1 vs. pT2-4; pN category, pN0-1a vs. N1b; Tumor size, <2cm vs. ≥2cm

Data are presented as rho correlation coefficients calculated by Spearman's rank correlation test and p values in parentheses; Rho coefficients exceeding 0.5 are shown in bold.

Supplementary Table S5. Univariate and multivariate Cox regression analysis for recurrence-free survival incorporating a 3% Ki-67 index cutoff as a high-grade criterion

Parameters	Comparison	HR	95% CI	p-value
Sex	Female vs. Male	3.569	1.028 – 12.388	0.045
Postoperative calcitonin	Continuous	1.002	1.000 - 1.003	0.013
pT2-4	pT1 vs. pT2-4	-	-	-
pN1b	pN0-1a vs. pN1b	42.077	5.131 - 345.059	<0.001
Grade incorporating a 3% Ki-67 index cutoff (hotspot)	Low grade vs. High grade	-	-	-
Multiplicity	Single vs. Multiple	-	-	-
Encapsulation	Present vs. Absent	-	-	-
Amyloid deposition/fibrosis	<50% vs. ≥50%	-	-	-
Lymphovascular invasion	Absent vs. Present	-	-	-
Gross extrathyroidal extension	Absent vs. present	-	-	-
Sex	Female vs. Male	4.714	1.284 – 17.306	0.019
Postoperative calcitonin	Continuous	1.002	1.000 - 1.003	0.019
pT2-4	pT1 vs. pT2-4	-	-	-
pN1b	pN0-1a vs. pN1b	35.245	4.179 - 297.271	0.001
Grade incorporating a 3% Ki-67 index cutoff (average)	Low grade vs. High grade	2.910	0.963 - 8.797	0.058
Multiplicity	Single vs. Multiple	-	-	-
Encapsulation	Present vs. Absent	-	-	-
Amyloid deposition/fibrosis	<50% vs. ≥50%	-	-	-
Lymphovascular invasion	Absent vs. Present	-	-	-
Gross extrathyroidal extension	Absent vs. Present	-	-	-

HR, hazard ratio; CI, confidence interval.

Supplementary Figure 1.

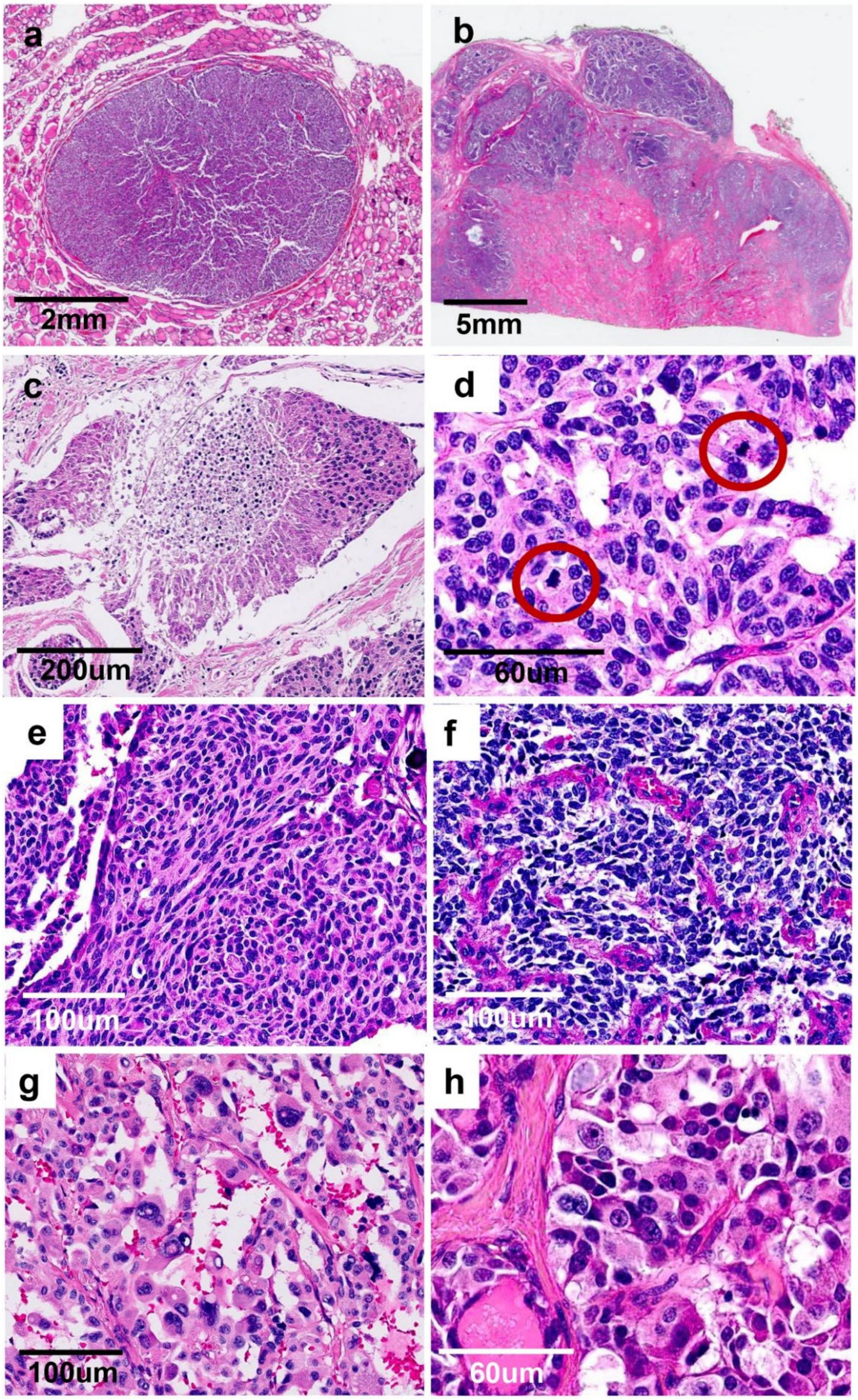

Supplementary Fig. 1 Representative histopathological features of medullary thyroid carcinoma. **a** An encapsulated tumor and **b** a tumor lacking encapsulation are depicted. **c** Tumor necrosis, **d** increased mitotic figures (red circle), **e** spindle cell morphology, **f** sheet-like growth pattern, **g** marked nuclear pleomorphism and **h** prominent nucleoli are observed.

Supplementary Figure 2.

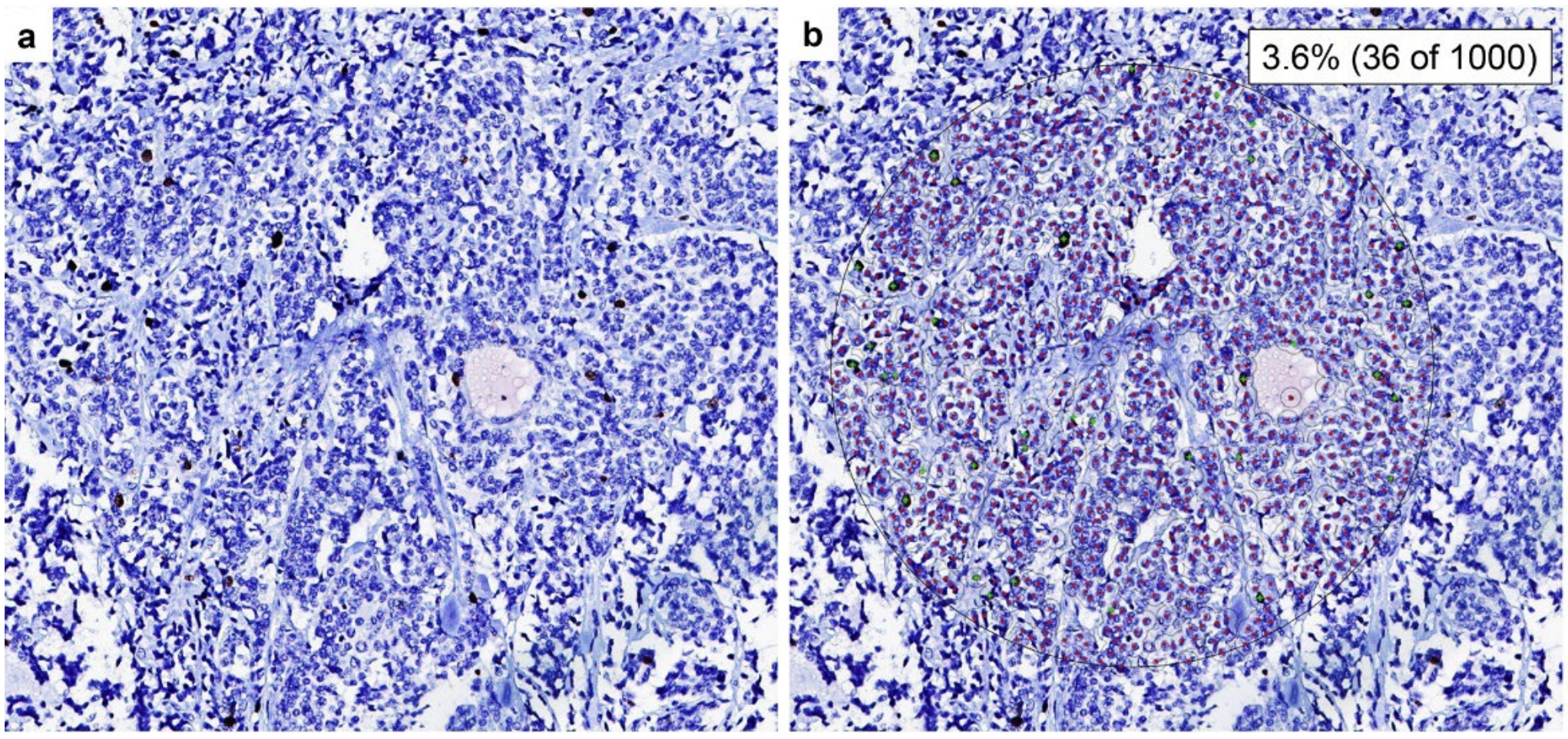

Supplementary Fig. 2 Digital quantification of the Ki-67 proliferation index in medullary thyroid carcinoma using the Sectra scoring application. **a** The unannotated image shows the field selected for Ki-67 assessment. **b** The same field is shown after application of the automated nuclear detection algorithm within a circular region of interest (ROI). Each detected tumor nucleus is annotated by the software: positive and negative nuclei are marked in green and red, respectively. The Ki-67 labeling index, displayed in the upper right corner, is calculated automatically as the proportion of positive nuclei among all detected tumor nuclei within the ROI. A minimum of 1000 tumor nuclei were assessed per ROI.
